# Editorial: Advances in rehabilitation intervention after limb amputation

**DOI:** 10.3389/fresc.2023.1149001

**Published:** 2023-02-16

**Authors:** David Crandell, Santiago Lozano-Calderon, Joel Mayerson

**Affiliations:** ^1^ Harvard Medical School, Spaulding Rehabilitation Hospital, Boston, United States; ^2^ Massachusetts General Hospital, Harvard Medical School, Boston, MA, United States; ^3^ Wexner Medical Center, The Ohio State University, Columbus, OH, United States

**Keywords:** rehabilitation, amputation, prosthetic rehabilitation, interdisciplinary team, prosthetic socket

**Editorial on the Research Topic** Advances in rehabilitation intervention after limb amputation

The tenth anniversary of the Boston Marathon Bombings is soon approaching on April 15, 2023. This mass casualty event highlighted the extraordinary role of bystanders and first responders who applied tourniquets, highly skilled trained trauma teams at the five Boston level I Trauma academic medical centers, followed by high quality, coordinated acute rehabilitation care in ensuring the best possible outcomes for the survivors (1–3). With this somber remembrance comes an opportunity to reflect on “lessons learned” during the past ten years by asking, “how far have we come?”

One way to address this question is to ask, “Are we better prepared today?” Those involved in the clinical care of the many seriously injured patients would agree that “the experience” did lead to several positive changes in the care of those with limb amputations. The advancement of multidisciplinary surgical care, combined with civilian and military training in acute care settings and high-quality rehabilitation, has become an integral part of the city's trauma care system. Access to advanced prosthetic componentry through “Boston Strong” community support has helped to change society's view of limb loss, destigmatizing those with limb differences.

When discussing these *Advances in Amputee Rehabilitation*, we like to consider the “3 T's”: Technology, Training, and Teams. Technology is “always improving, right?” Innovations in prosthetic technology are occurring at a rapid rate, especially user control, power, and biocompatibility. Cutting-edge advances in magnetomicrometry (4) and optogenetics (5) may further enhance myoelectric control. Providing net power at prosthetic joints may improve biomechanical function. Implantable devices allowing direct sensory feedback with prolonged biocompatibility may lead to true “bionic-integration (6).”

## Technology—socket fit

An uncomfortable socket remains the most common patient complaint today. The traditional prosthetic socket and liner technologies may impact the health of the residuum, especially in dysvascular amputees (7, 8). Khetarpual et al. *Frontiers | Issues Faced by Prosthetists and Physiotherapists During Lower-Limb Prosthetic Rehabilitation: A Thematic Analysis* highlight issues experienced by clinicians during lower-limb prosthetic rehabilitation. Their insights, gained from clinical participants and prosthesis users in their study provide a more complete view of the rehabilitation process following amputation. Their study reveals that even when the residual limb shape and volume have stabilized, unfortunately the tissue in the residual limb may not be suited for prosthesis use. Skin grafts in those patients with traumatic amputations can lead to adherent tissue with significant subsequent pain and skin breakdown. In the research presented by Olsen et al. communication between patients and their clinicians was truly important in evaluating socket comfort *Frontiers | The Impact of Limited Prosthetic Socket Documentation: A Researcher Perspective*. They noted that the terms “socket fit” and “comfort” were used interchangeably; socket fit assessed by the prosthetist focus is on the volume, shape, and suspension of the residual limb, whereas socket comfort is a subjective measure reported by the prosthesis user. Outlining guidelines for socket fit quality may lead to a universally accepted outcome measures, capable of enhancing communication and data-sharing between patients, clinicians, and researchers, along with participation in the National Amputee Registry (9).

## Training—surgery / rehabilitation

Efforts to incorporate newer technical developments in amputation surgery and reconstruction demonstrate a shift toward an outcomes-oriented approach in amputee care (10–13). Surgical training in advanced and modified techniques for amputations has expanded to include osteomyoplasty and osseo-integration, agonist-antagonist myoneural interfaces (AAMIs), tibia-turnup plasty, rotationplasty, targeted muscle reinnervation (TMR) and regenerative peripheral nerve interfaces (RPNIs). The latter two address some of the deleterious postoperative sequelae associated with amputation (namely, chronic limb pain and neuroma formation), which may limit prosthetic compatibility and use. Shotande et al. *Frontiers | Comparing Temporospatial Performance During Brisk and Self-Paced Walking by Men With Osteomyoplastic Transfemoral Amputation and Controls Using Pressure and Muscle Activation Peak Times* studied residuum-socket interface (RSI) pressures and residuum muscle activation after transfemoral osteomyoplastic amputations (OTFA) during brisk and self-paced gait compared to the performance with that of intact controls. They showed that RSI pressures were distributed throughout the residuum-socket and that muscles were engaged and often co-contracted at key times during the gait cycle. Their recommendations include enhancing specific therapeutic exercises as part of OTFA rehabilitation to improve control activation of hip adductors, hamstrings, and quadriceps in the intact and distal-residual limbs, thereby optimizing overall gait performance stability and reducing excessive energy expenditure. This study highlights that new reconstruction techniques require that new, patient-centered rehabilitation protocols be developed and studied.

## Team—it takes a team

When treating individuals with severe limb trauma, important decisions around limb salvage vs. amputation and optimal amputation level are ideally made through an interdisciplinary approach to optimize both surgical and functional outcomes (14). The last research article by Khetarpaul et al. focuses on the interdisciplinary team approach (15) as the model system of amputee care. *Frontiers | Socioecological model-based design and implementation principles of lower limb preservation programs as partners for limb-loss rehabilitation programs—A mini-review*. Their review article can be helpful to healthcare institutions and organizations seeking to develop, expand, or refine their ability to provide comprehensive limb care. The model system of care includes presurgical planning, postsurgical and early pre-prosthetic rehabilitation care, and prosthetic and lifelong care. Rehabilitation care includes coordination with community resources, such as peer support, and vocational, recreational, and driving assessment. The emphasis on prior mental health concerns and new concerns that arise with limb loss should also be addressed by mental health professionals in the multidisciplinary care provider team model.

We are confident that this series of articles will advance the multidisciplinary surgical and rehabilitation care that takes place before and after limb amputation and will provide a successful framework for ongoing research.
